# Supplementary material for: Epigenetic inactivation of the autophagy–lysosomal system in appendix in Parkinson’s disease
Source: Nat Commun. 2021 Aug 26;12:5134. doi: 10.1038/s41467-021-25474-x (PMC8390554; doi:10.1038/s41467-021-25474-x)
Supplement: Supplementary file 1 — Supplementary Information [file 41467_2021_25474_MOESM1_ESM.pdf]

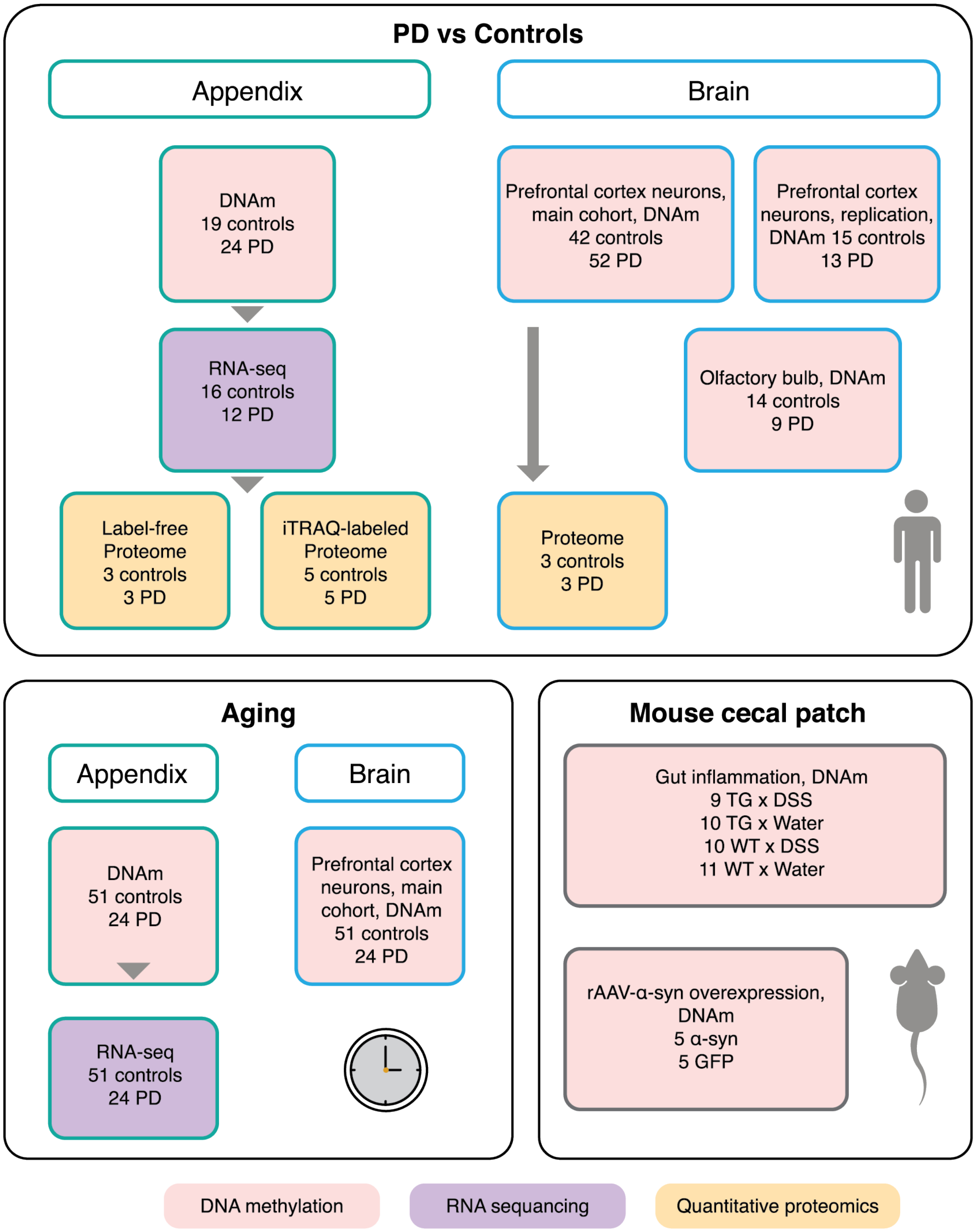

Supplementary Figure 1. Overview of study design, including sample cohorts and experiments conducted. Detailed information on each sample cohort in Supplementary Data 18. Gray arrow indicates subsequent analysis performed on a sample subset.

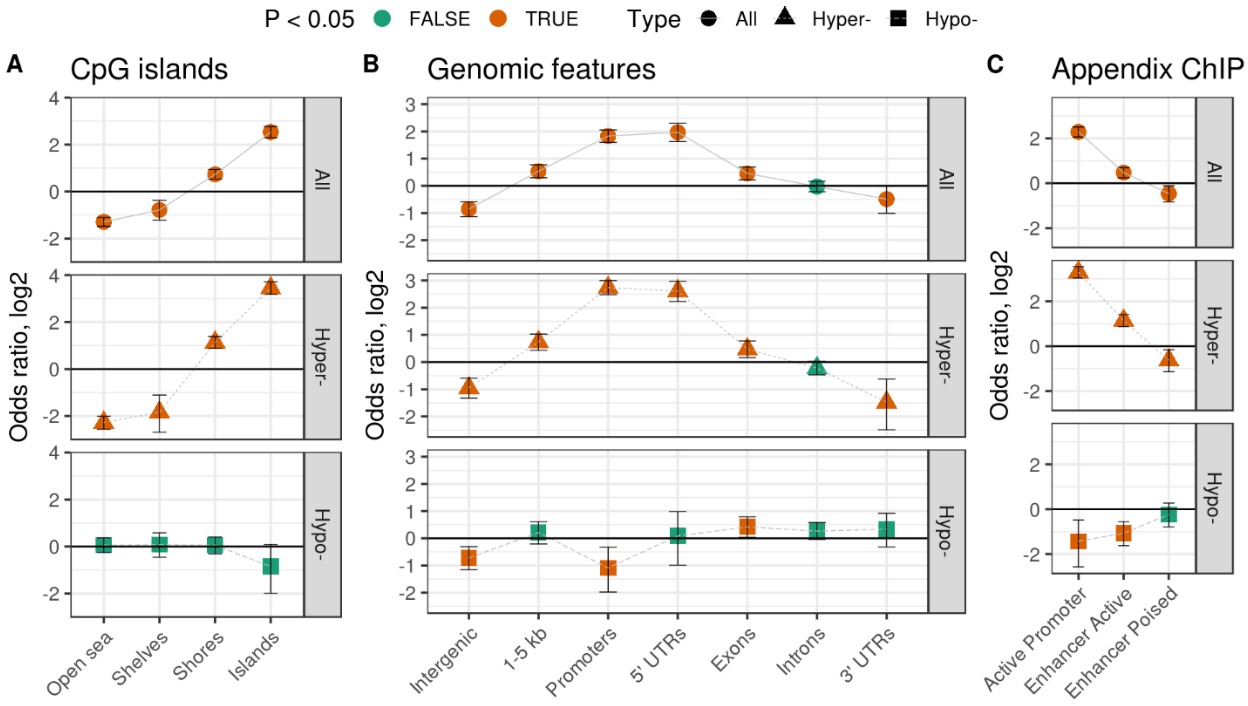

Supplementary Figure 2. Genomic elements enriched with differentially methylated cytosines in the PD appendix. (**A**, **B**, and **C**) Genomic elements in the PD appendix enriched with differentially methylated cytosines (All, upper row), hypermethylated cytosines (Hyper-, middle row), and hypomethylated cytosines (Hypo-, lower row), relative to the healthy control appendix (*n* = 24 PD and 19 controls). CpG island and genomic features determined by Bioconductor annotatr package [130] and active/poised enhancers and promoters in the appendix determined by ChIP-seq of H3K4me1 and H3K27ac chromatin marks in appendix. Orange circles represent $p < 0.05$, two-sided Fisher's exact test. Error bars indicate 95% confidence intervals.

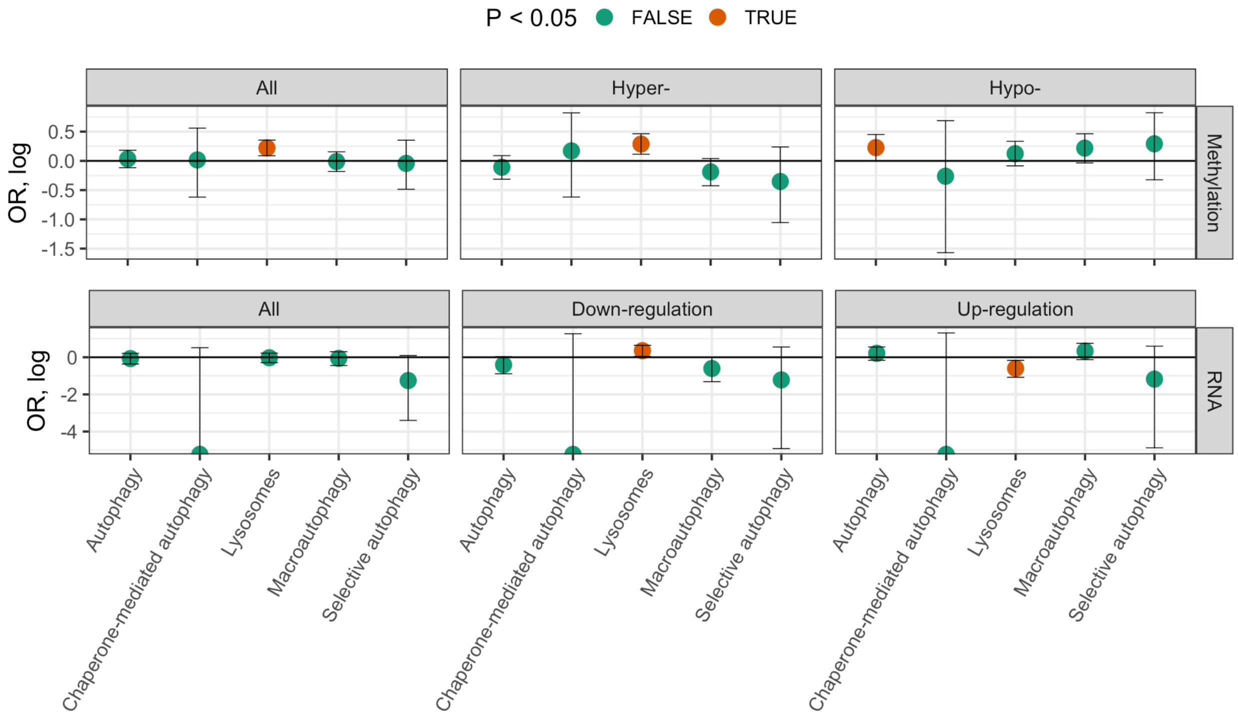

Supplementary Figure 3. ALP pathway changes in DNA methylation and gene expression in the PD appendix. ALP pathways enriched with differential methylation (upper row) and differentially expressed genes (lower row) in the PD appendix. ALP pathways affected by DNA methylation changes in PD patients relative to controls were determined (n = 24 PD and 19 controls) and compared to transcriptionally altered pathways (n = 16 controls and 12 PD). Shown are all (left), hypermethylated/transcriptionally downregulated (middle), and hypomethylated/transcriptionally upregulated (right) pathways. Orange circles represent $p < 0.05$, two-sided Fisher's exact test. Error bars indicate 95% confidence intervals.

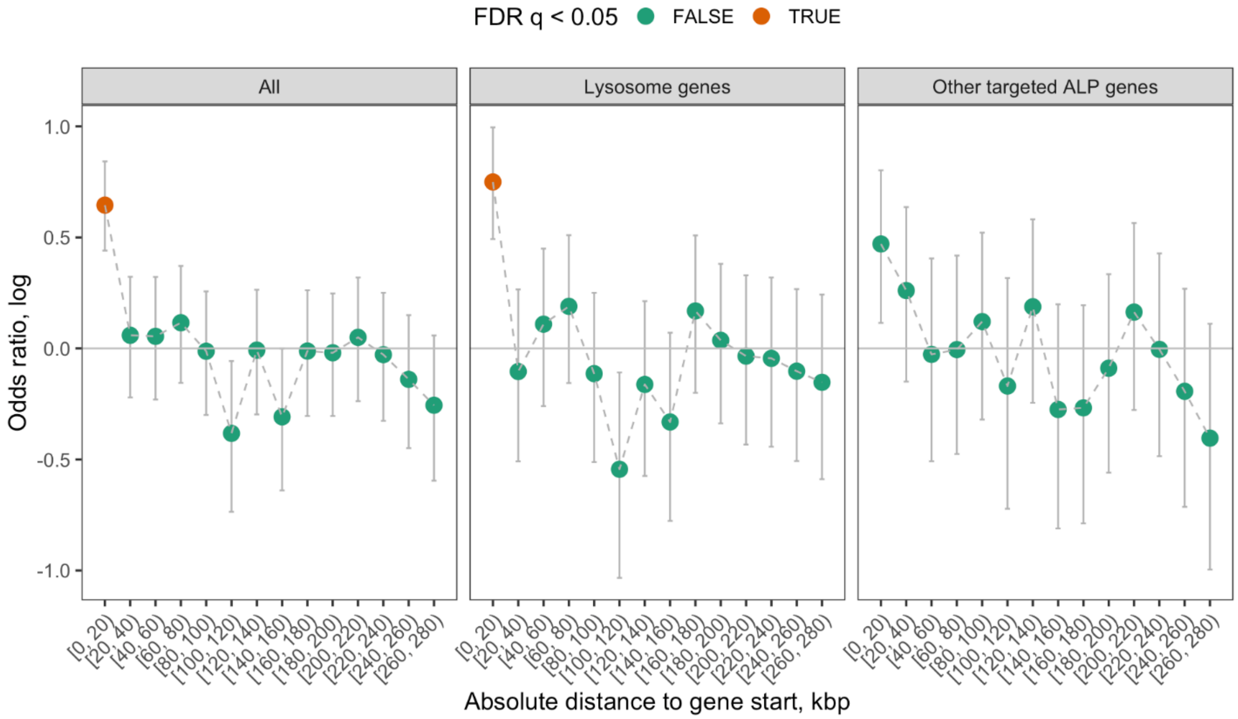

Supplementary Figure 4. Proximity of DNA methylation changes to ALP genes. Enrichment of ALP genes with differentially methylated cytosines in the PD appendix as a function of distance from gene transcription start site. All the genes targeted in DNA methylation analysis (left panel) were stratified into those belonging to lysosome pathway (middle panel) and the rest (other targeted ALP genes, right panel). Every CpG site was assigned a 20 kb-wide bin based on the distance to the transcription start site of the nearest ALP gene. For each gene strata, the odds of observing differentially methylated cytosines within a given bin were compared to the odds of observing differentially methylated cytosines outside of the bin using two-sided Fisher's exact test and resulting *p*-values were FDR corrected. Error bars indicate 95% confidence intervals computed using n = 177,145, n = 95,962 and n = 81,183 cytosines for all, lysosome and other targeted ALP genes, respectively.

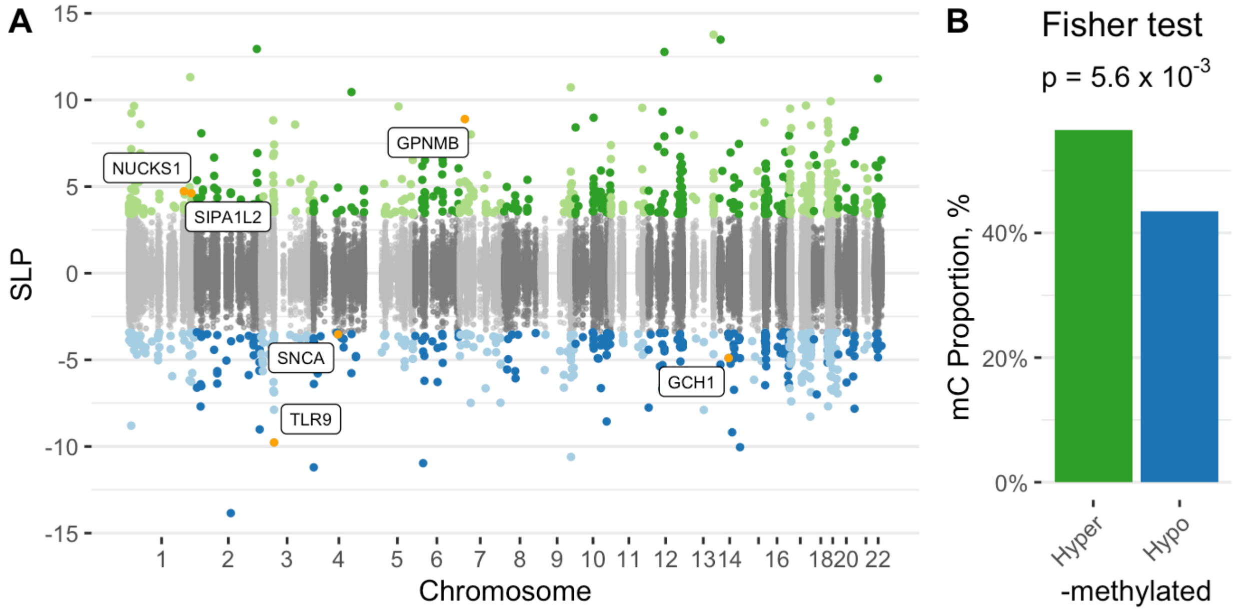

Supplementary Figure 5. DNA methylation changes in olfactory bulb of PD patients and controls. (**A**) Manhattan plot demonstrating differential methylation in PD olfactory bulb (1,142 cytosines at FDR $q < 0.05$, robust linear regression; $n = 9$ PD and 14 controls). Highlighted are genes implicated in both our study and PD GWAS [23]. SLP refers to signed log *p*-value, with sign corresponding to the direction of DNA methylation change. (**B**) Fraction of significantly hypermethylated (green) and hypomethylated (blue) sites in the PD olfactory bulb. The *p*-value represents two-sided Fisher's exact test for enrichment of hypermethylated sites among significant versus non-significant cytosines.

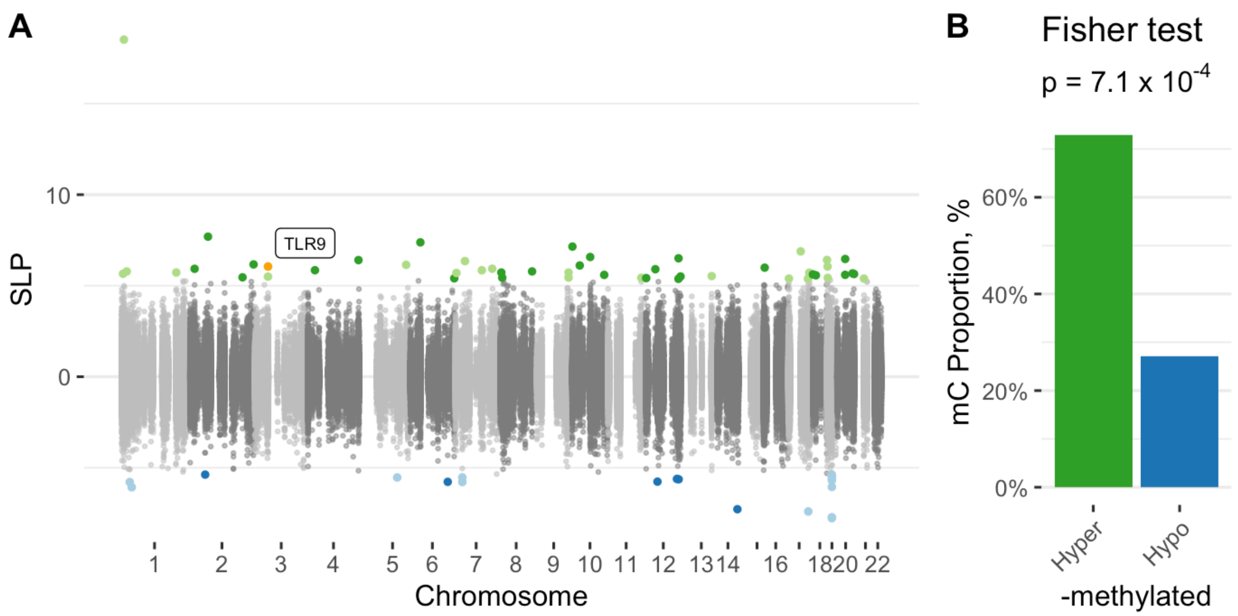

Supplementary Figure 6. DNA methylation changes in prefrontal cortex neurons of PD patients and controls. (**A**) Manhattan plot demonstrating differential methylation in PD prefrontal cortex neurons (70 cytosines at $q < 0.05$, robust linear regression; $n = 52$ PD and 42 controls). Highlighted are genes implicated in both our study and PD GWAS [23]. SLP refers to signed log *p*-value, with sign corresponding to the direction of DNA methylation change. (**B**) Fraction of significantly hypermethylated (green) and hypomethylated (blue) sites in the PD prefrontal cortex neurons. The *p*-value represents two-sided Fisher's exact test for enrichment of hypermethylated sites among significant versus non-significant cytosines.

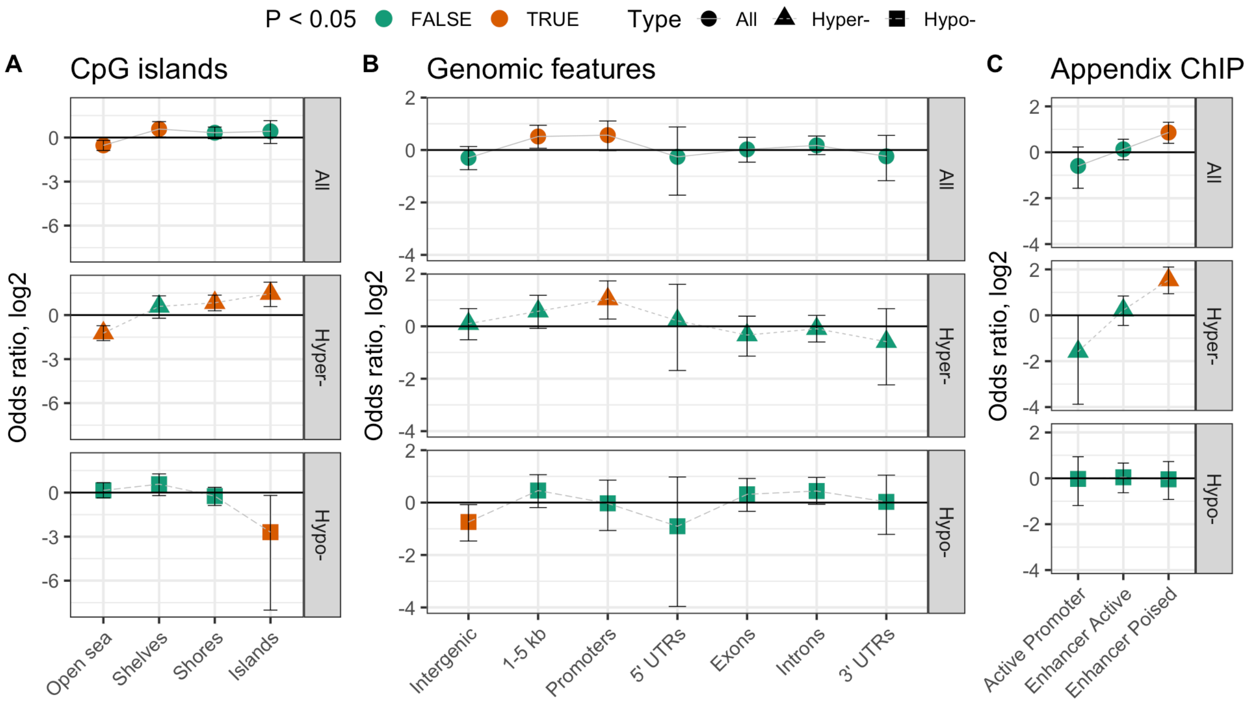

Supplementary Figure 7. Genomic elements enriched by cytosines differentially methylated with age in the appendix of healthy controls. (**A**, **B**, and **C**) Genomic elements enriched with age affected cytosines (All, upper row), hypermethylated cytosines (Hyper-, middle row), and hypomethylated cytosines (Hypo-, lower row) (*n* = 51 individuals). CpG island and genomic features determined by Bioconductor annotatr package [130] and active/poised enhancers and promoters in the appendix determined by ChIP-seq of H3K4me1 and H3K27ac chromatin marks in appendix. Orange circles represent $p < 0.05$, two-sided Fisher's exact test. Error bars indicate 95% confidence intervals.

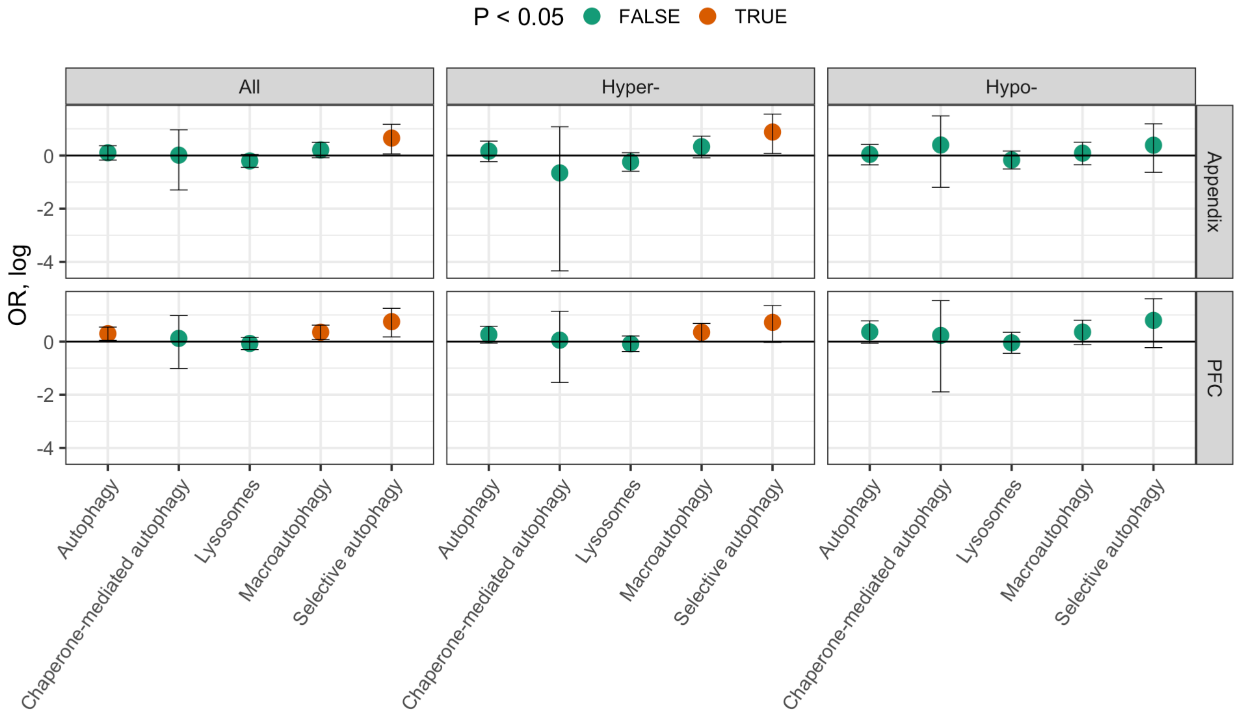

Supplementary Figure 8. Age related DNA methylation changes of ALP pathways in the healthy appendix and prefrontal cortex neurons. ALP pathways enriched with significant methylation age effects in the healthy appendix (upper row) and prefrontal cortex neurons (lower row). ALP pathways affected by DNA methylation changes with age were examined in the appendix and prefrontal cortex neurons of healthy control individuals (n = 51 and 42 individuals, respectively). Shown are all (left), hypermethylated (middle), and hypomethylated (right) pathways. Orange circles represent $p < 0.05$, two-sided Fisher's exact test. Error bars indicate 95% confidence intervals.

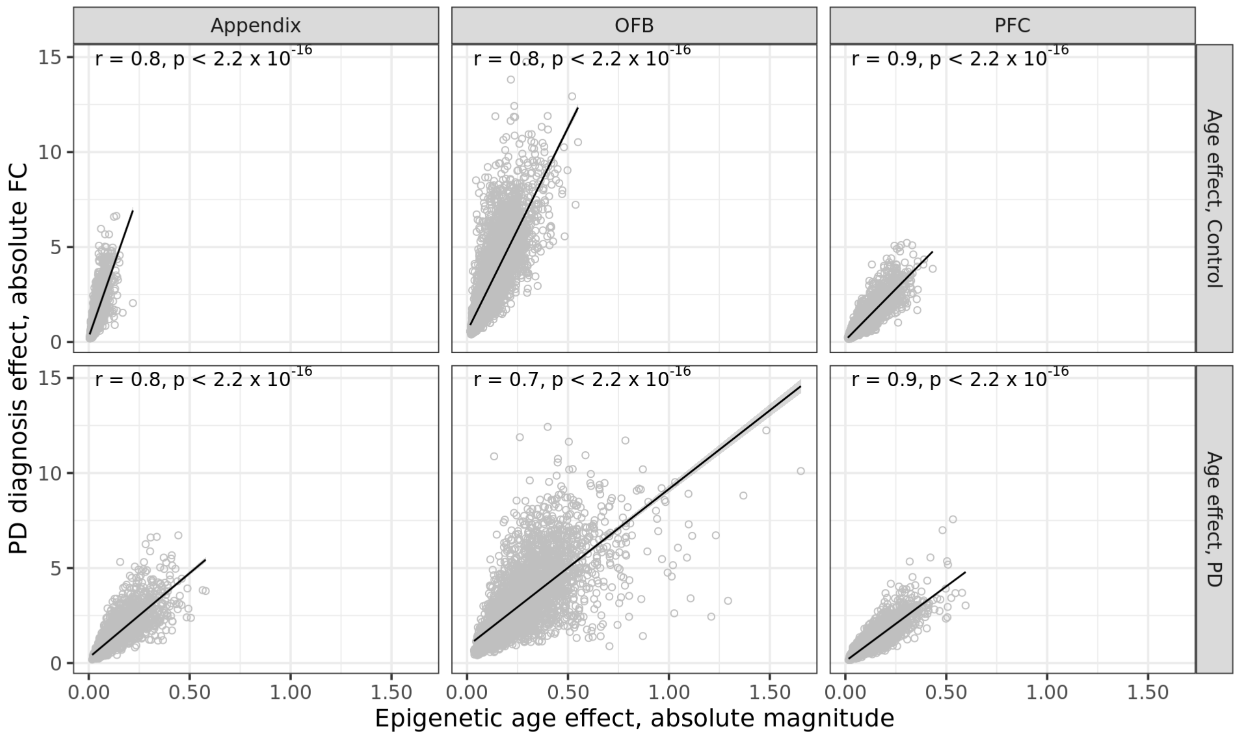

Supplementary Figure 9. The relationship between epigenetic changes occurring with age and epigenetic changes occurring in PD, examining the appendix, olfactory bulb and prefrontal cortex neurons among healthy and PD samples. Each point represents a cytosine having a nominally significant (two-sided $p < 0.05$) methylation change in PD and nominally significant age effect in control or PD group. Correlation coefficients and their statistical significance determined using Pearson correlation. Appendix n = 31 controls, 24 PD; prefrontal cortex n = 35 controls, 52 PD; olfactory bulb n = 14 controls, 9 PD.

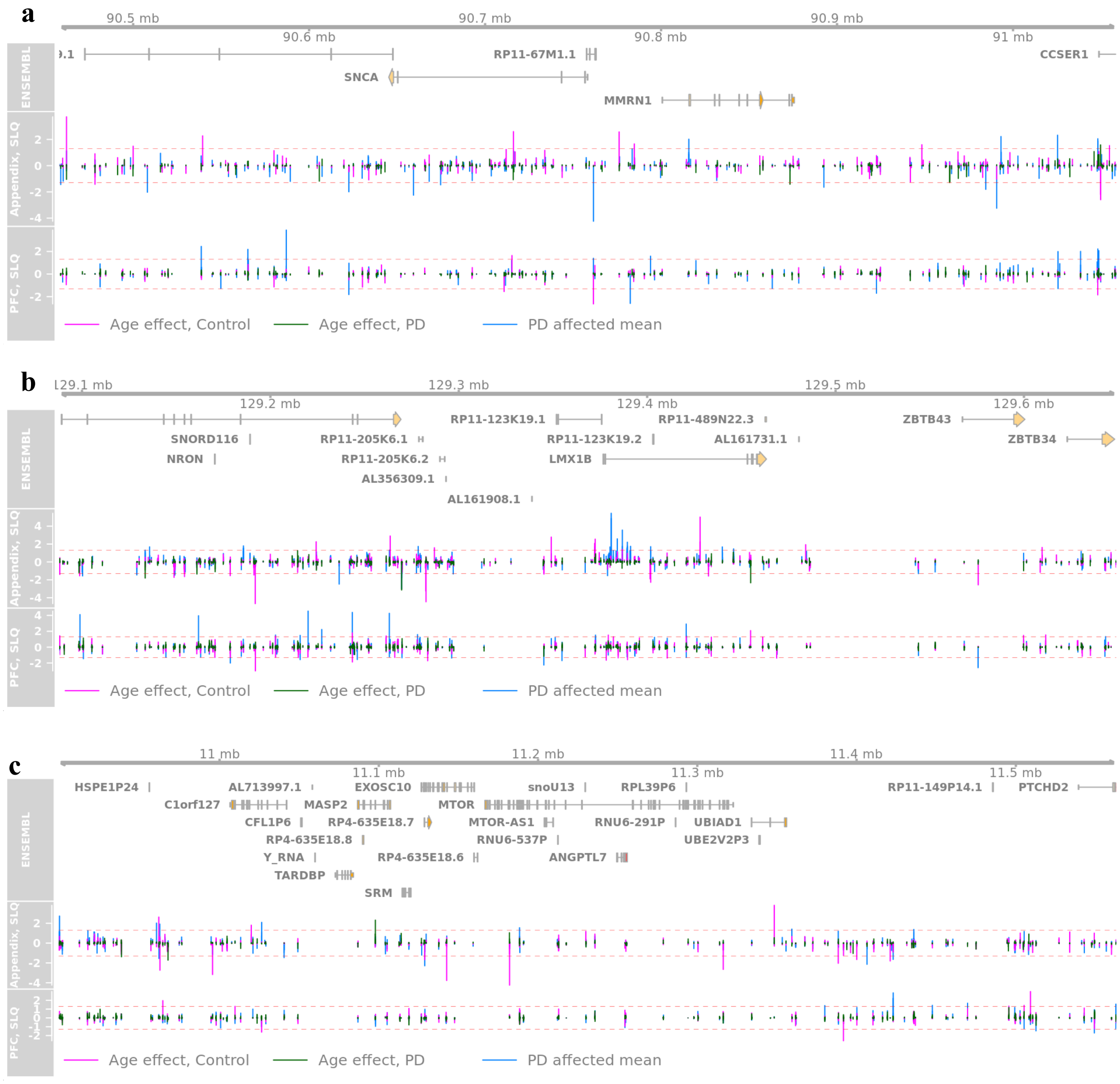

Supplementary Figure 10. PD related changes and age effects at individual cytosines surrounding PD implicated genes in PD appendix and prefrontal cortex neurons. (**a**) *SNCA*. (**b**) *LMX1B*. (**c**) *MTOR*. Purple and green bars show methylation changes with age in control and PD, respectively, and blue bars show methylation changes in PD compared to control. Bar heights correspond to signed log-transformed FDR q value. Red dashed lines indicate FDR $q < 0.05$ threshold. Transparent filled rectangles indicate the area corresponding to the gene transcript.

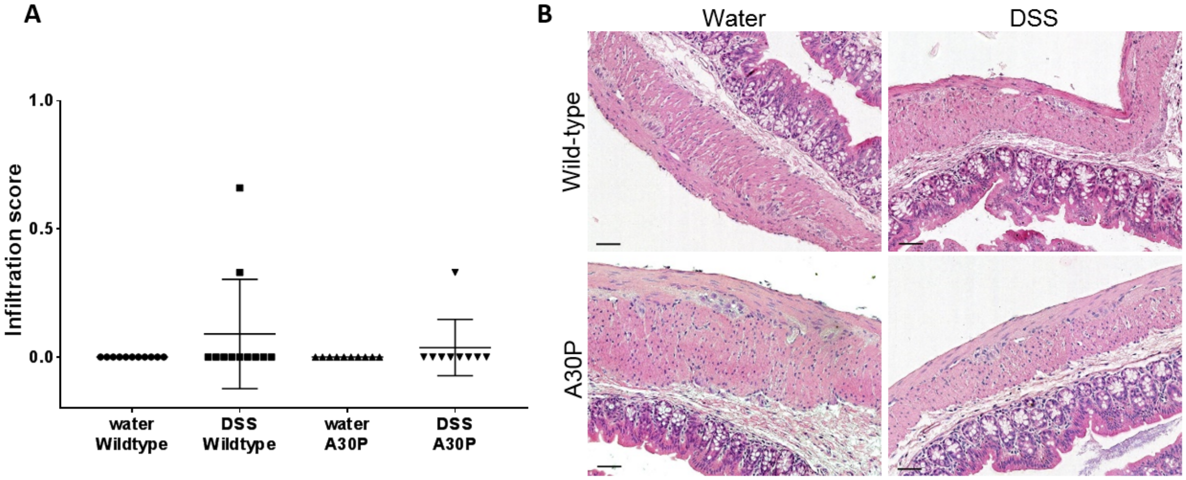

Supplementary Figure 11. Histological analysis of the gut of wild-type and A30P α-syn mice previously exposed to DSS colitis. After the recovery period, mice that experienced DSS colitis have a histologically normal colon, as determined by leukocyte infiltration and tissue integrity. Wild-type and transgenic A30P α-syn mice were exposed to DSS colitis or remained on normal drinking water, followed by a 4-week recovery period. (**A**, **B**) Inflammation and tissue integrity in colon of wild-type and A30P α-syn mice at the end of the recovery period was analyzed in 10 μm thick paraffin sections processed for hematoxylin/eosin staining. *n* = 9 A30P/DSS colitis, 10 A30P/Water, 10 WT/DSS colitis, 11 WT/Water. Data are presented as mean values +/- SD. Scale bar is 50 μm.

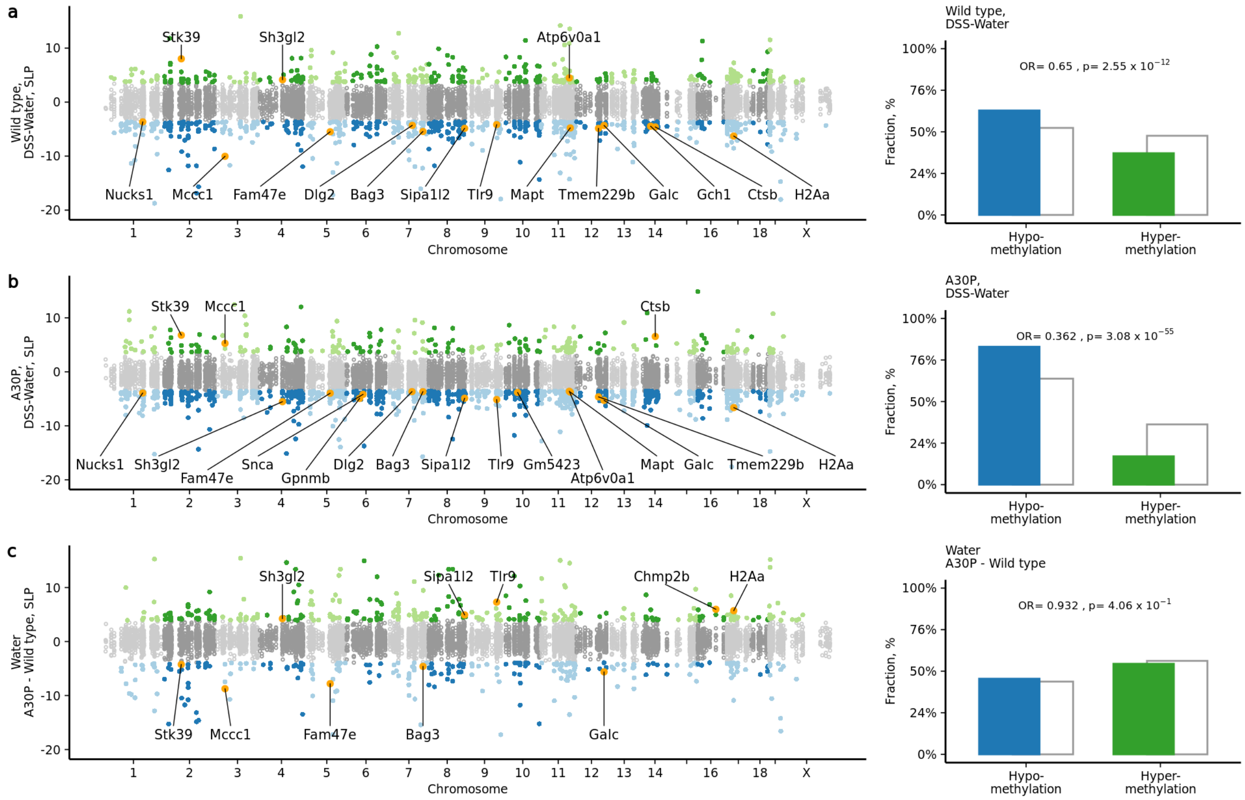

Supplementary Figure 12. Differential methylation of the ALP in the cecal patch of A30P α-syn mice. DNA methylation was profiled at 571 ALP genes in the cecal patch of mice overexpressing human α-syn with the heterozygote A30P mutation (A30P α-syn mice), a PD-relevant model of synucleinopathy. (**a**) In wild-type mice that experienced DSS-mediated gut inflammation there were 397 genes in the ALP exhibiting differential methylation relative to wild-type mice that did not experience DSS colitis (1,104 sites at $q < 0.05$, robust linear regression). (**b**) In A30P α-syn mice that experienced DSS-mediated gut inflammation there were 408 genes in the ALP exhibiting differential methylation relative to A30P mice that did not experience DSS colitis (1,378 sites at $q < 0.05$, robust linear regression). (**c**) In A30P α-syn mice treated with water there were 315 genes in the ALP exhibiting differential methylation relative to wild-type mice treated with water (591 sites at $q < 0.05$, robust linear regression). ALP genes implicated in both our study and PD risk by GWAS [23] (mapped to corresponding mouse genes) are labeled. SLP refers signed log *p*-value, with sign corresponding to the direction of DNA methylation change (hypermethylation, green or hypomethylation, blue). Barplots indicate the fraction of significantly hypermethylated (green) and hypomethylated (blue) sites. The enrichment of hypermethylated loci was determined by two-sided Fisher's exact test, OR < 1 indicates dominant hypomethylation.

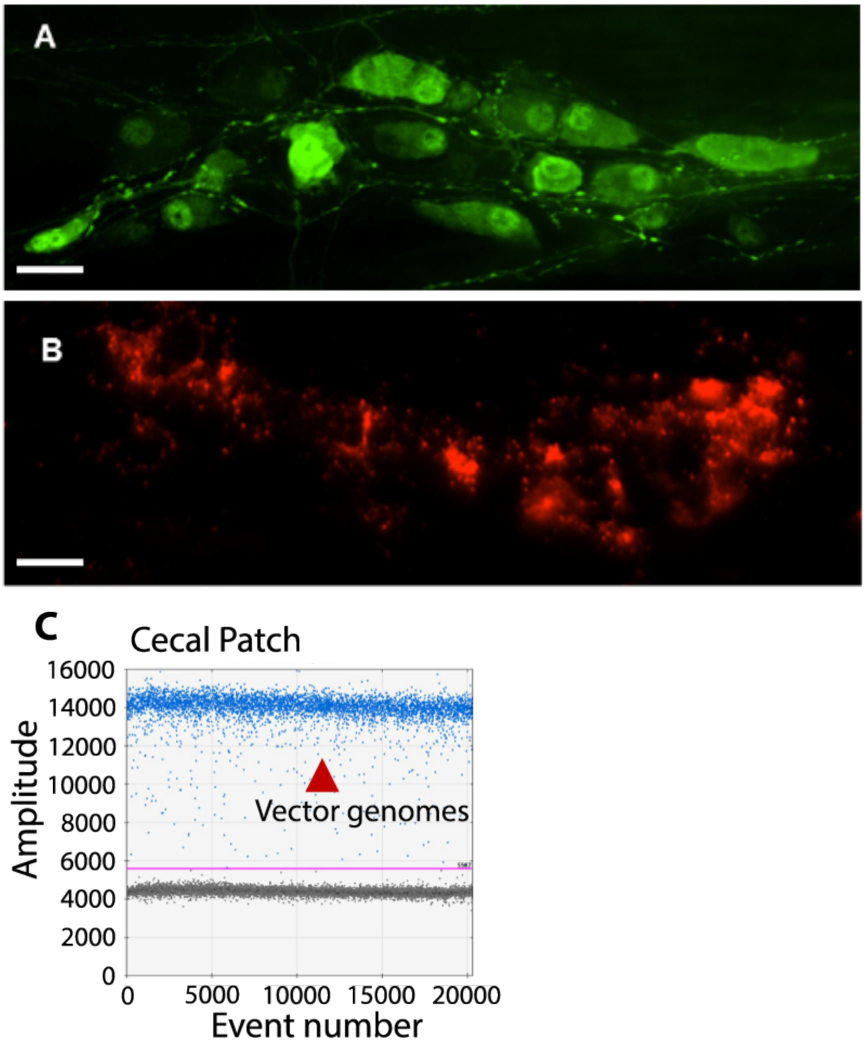

Supplementary Figure 13. rAAV-mediated human α-syn overexpression in myenteric neurons of the mouse cecal patch results in α-syn aggregation. A rAAV vector, expressing human α-syn specifically in neurons, was injected subserosally into the cecal patch of C57BL/6J mice (*n* = 10) and α-syn pathology was examined 1 month later in all animals not utilized for DNA methylation analysis (*n* = 5). rAAV-mediated overexpression of human α-syn gives rise to a high degree of α-syn aggregates in enteric neurons of myenteric plexi of the mouse cecal patch. (**A**) Punctate human α-syn (green) was apparent in cecal patch neurons, signifying robust α-syn overexpression with the rAAV approach. Scale bar is 20 μm. (**B**) Aggregated α-syn positive for phosphor-serine 129 (red, 81A antibody) in enteric neurons of the mouse cecal patch. Scale bar is 20 μm. (**C**) Digital droplet PCR analysis of rAAV vector genomes in tissue isolated from the mouse cecal patch, demonstrating successful delivery of rAAV.

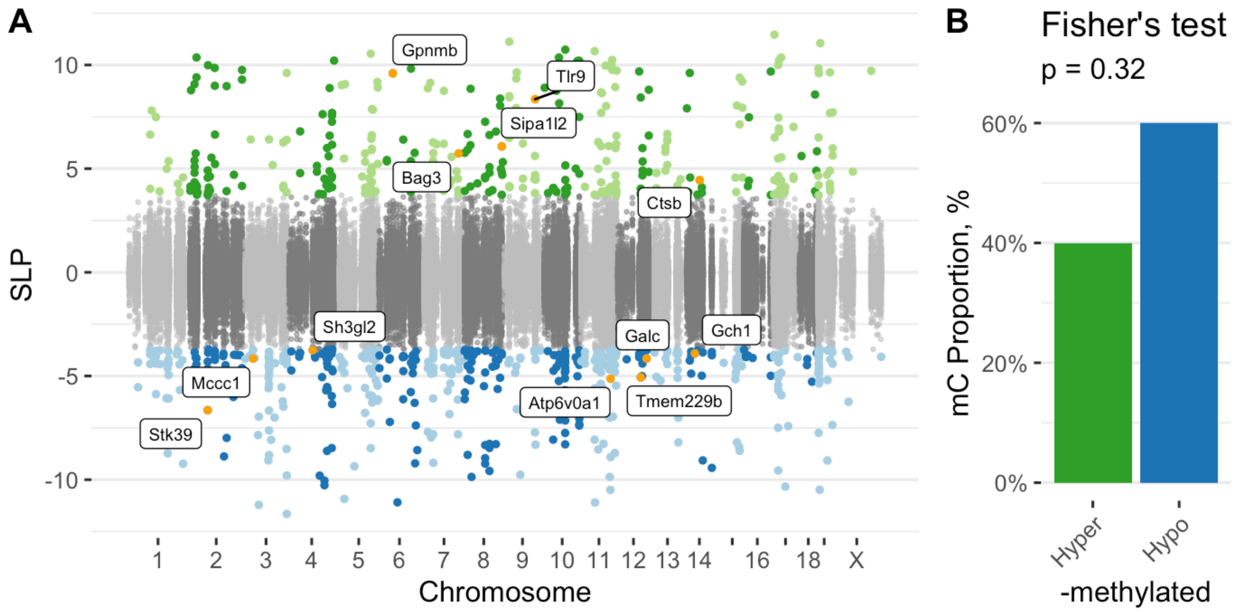

Supplementary Figure 14. Differential methylation of the ALP in the cecal patch of mice with α-syn aggregation. (**A**) Manhattan plot demonstrating differentially methylated cytosines at ALP genes in the cecal patch of mice with rAAV-mediated α-syn aggregation ($q < 0.05$, robust linear regression; $n = 5$ mice with rAAV-α-syn vector and 5 mice with rAAV-GFP control vector; C57BL6/J mice). Highlighted are ALP genes implicated in both our study and PD risk by GWAS [23] (mapped to corresponding mouse genes). SLP refers to signed log p-value, with sign corresponding to the direction of DNA methylation change. (**B**) Fraction of significantly hypermethylated (green) and hypomethylated (blue) sites in the cecal patch of mice with rAAV-induced α-syn overexpression. The p-value was determined by two-sided Fisher's exact test.

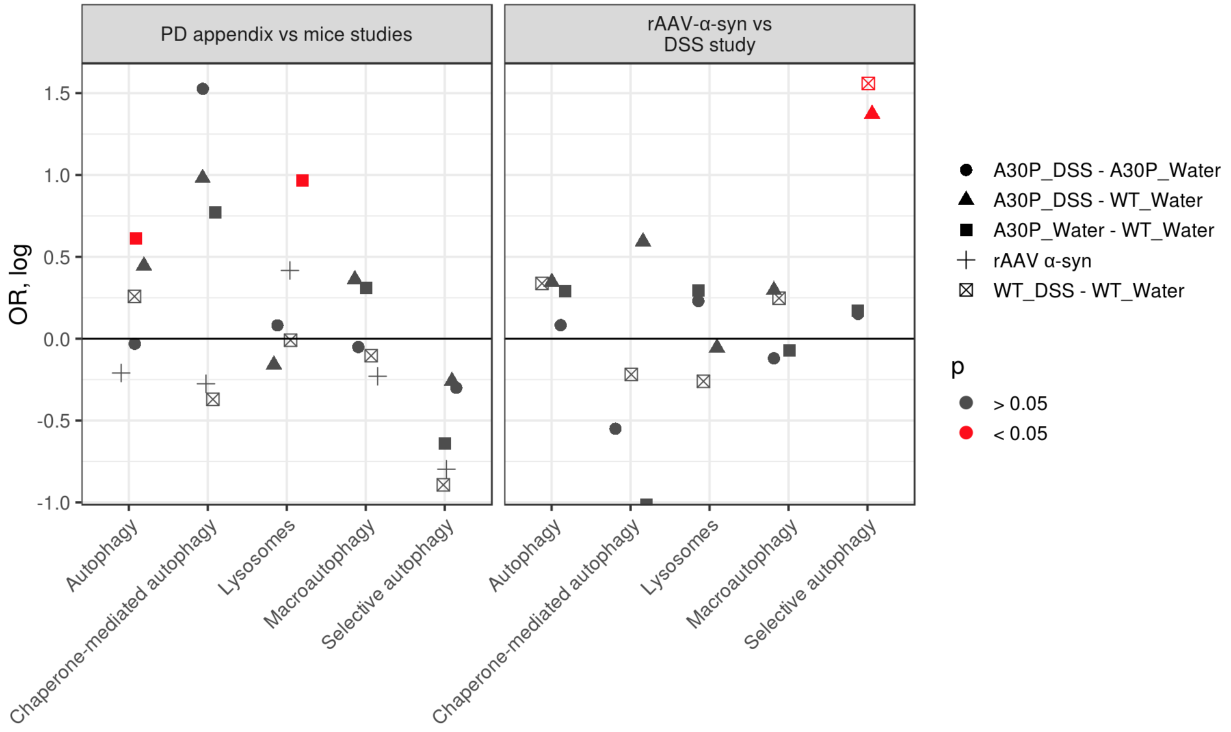

Supplementary Figure 15. ALP pathway changes in the PD appendix compared to the mouse studies. ALP pathway enrichment for genes epigenetically dysregulated in both the PD appendix and in the mice studies. The left panel shows ALP pathways that are epigenetically disrupted in both the appendix of PD patients and the murine appendix (cecal patch) of the gut inflammation model or α-syn aggregation model (PD appendix, *n* = 24 PD and 19 controls; gut inflammation model, *n* = 9 A30P/DSS colitis, 10 A30P/Water without colitis, 10 WT/DSS colitis, 11 WT/Water without colitis; α-syn aggregation model, *n* = 5 control vector and 5 α-syn overexpression vector). The right panel shows ALP pathways affected by epigenetic changes in both the α-syn aggregation model and the gut inflammation model in mice. Red color ($p < 0.05$, two-sided Fisher's exact test) shows significant ALP pathway enrichment between comparison groups (PD appendix *vs.* mouse groups, or α-syn aggregation *vs*. gut inflammation mouse groups).

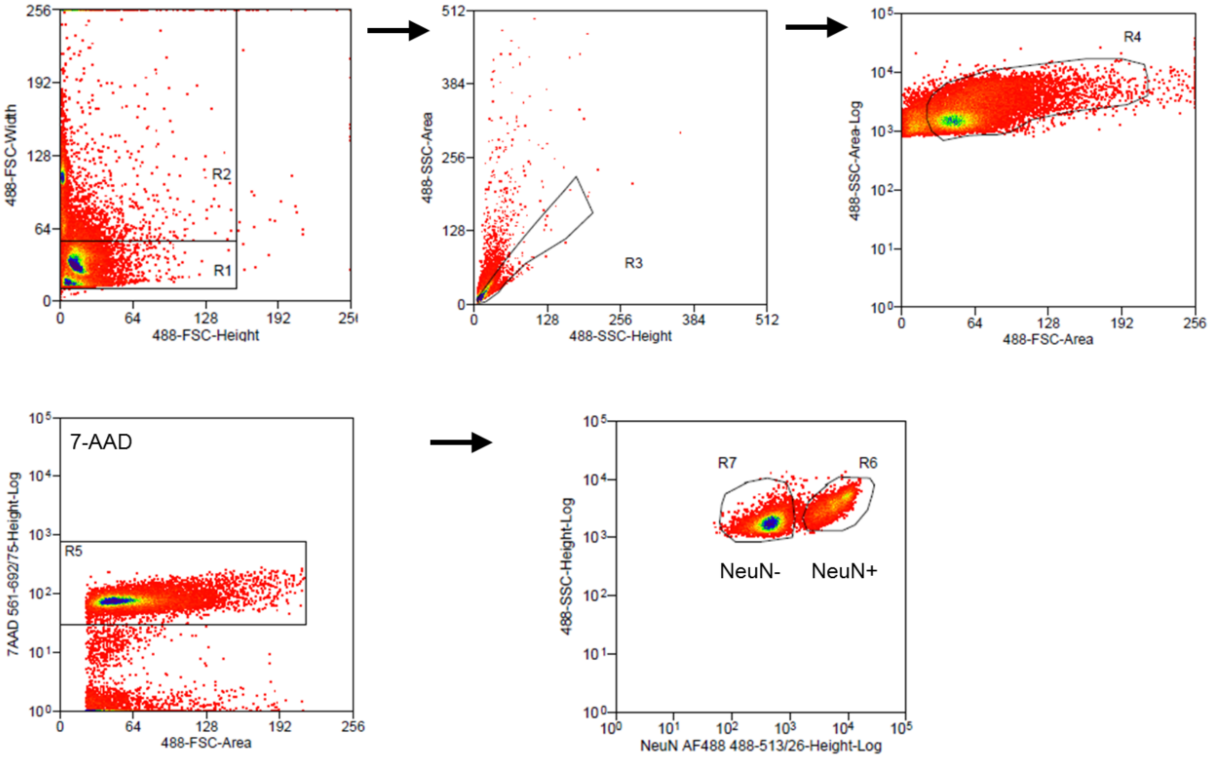

Supplementary Figure 16. Flow cytometry gating strategy for neuronal nuclei isolation from prefrontal cortex. Neuronal nuclei were isolated from the prefrontal cortex of PD patients and controls using a flow cytometry-based approach, as described [52, 111]. Briefly, human brain tissue (~250 mg) for each sample was minced in 2 mL PBSTA (0.3 M sucrose, 1X phosphate buffered saline (PBS), 0.1% Triton X-100) and homogenized in PreCellys CKMix tubes with a Minilys (3,000 rpm for 5 s, 5 min on ice, 3 times) (Bertin Instruments). Sample homogenates were filtered through Miracloth (EMD Millipore), rinsed with 2 mL of PBSTA and placed on a sucrose cushion (1.4 M sucrose). Nuclei were pelleted by centrifugation at 4000 × *g* for 30 min 4°C using a swinging bucket rotor, and the pellet was incubated in 700 µl of 1X PBS on ice for 20 min. The nuclei were gently resuspended and blocking mix (100 µl of 1X PBS with 0.5% BSA (Thermo Fisher Scientific), and 10% normal goat serum (Gibco)) was added to each sample. Anti-NeuN antibody with Alex Fluor 488 (1:500; Abcam; ab190195) was added and samples were incubated 45 min at 4°C with gentle mixing. Immediately prior to flow cytometry sorting, nuclei were stained with 7-AAD (Thermo Fisher Scientific) and passed through a 30 µM filter (SystemX). Neuronal nuclei (NeuN and 7-AAD stained) were isolated using a MoFlo Astrios (Beckman Coulter) running Summit 6.3 by the Van Andel Research Institute Flow Cytometry Core. Approximately 1 million NeuN+ nuclei were sorted for each sample with an average purity of 97%.

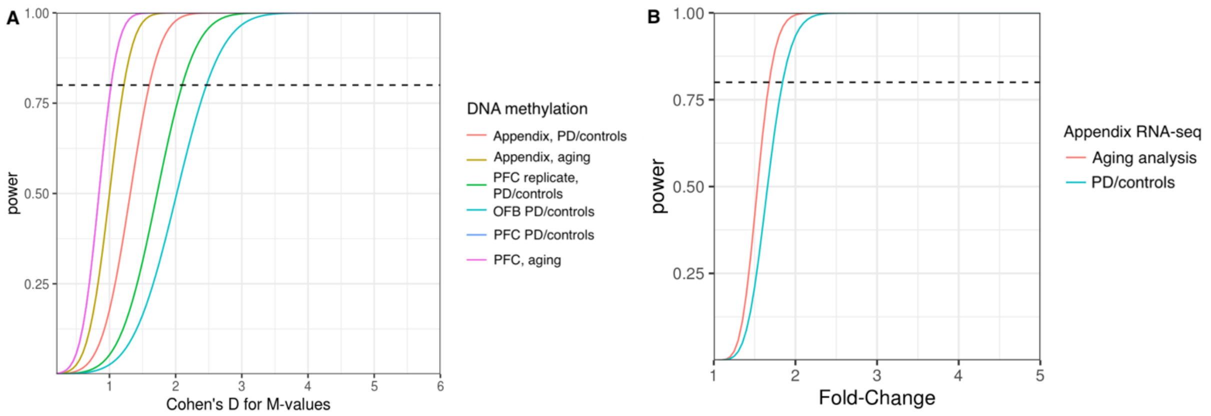

Supplementary Figure 17. Estimation of statistical power to detect varying effect sizes for datasets in this study. Statistical power to detect varying effect sizes was estimated using the R power.t.test function for bisulfite padlock probe sequencing data and the Bioconductor RNASeqPower function for RNA-seq data. (**A**) Power estimation for DNA methylation datasets computed conservatively assuming FDR adjusted alpha of 0.0001. Cohen's D, the ratio of fold change to standard deviation, is used as measure of effect size. The dashed line at 0.8 indicates that 80% of true positives exhibiting corresponding effect size will be identified. (**B**) Power estimation for RNA-seq datasets computed conservatively assuming FDR adjusted alpha of 0.0001, biological coefficient of variation = 0.3. The dashed line at 0.8 indicates that 80% of true positives exhibiting corresponding effect size will be identified.

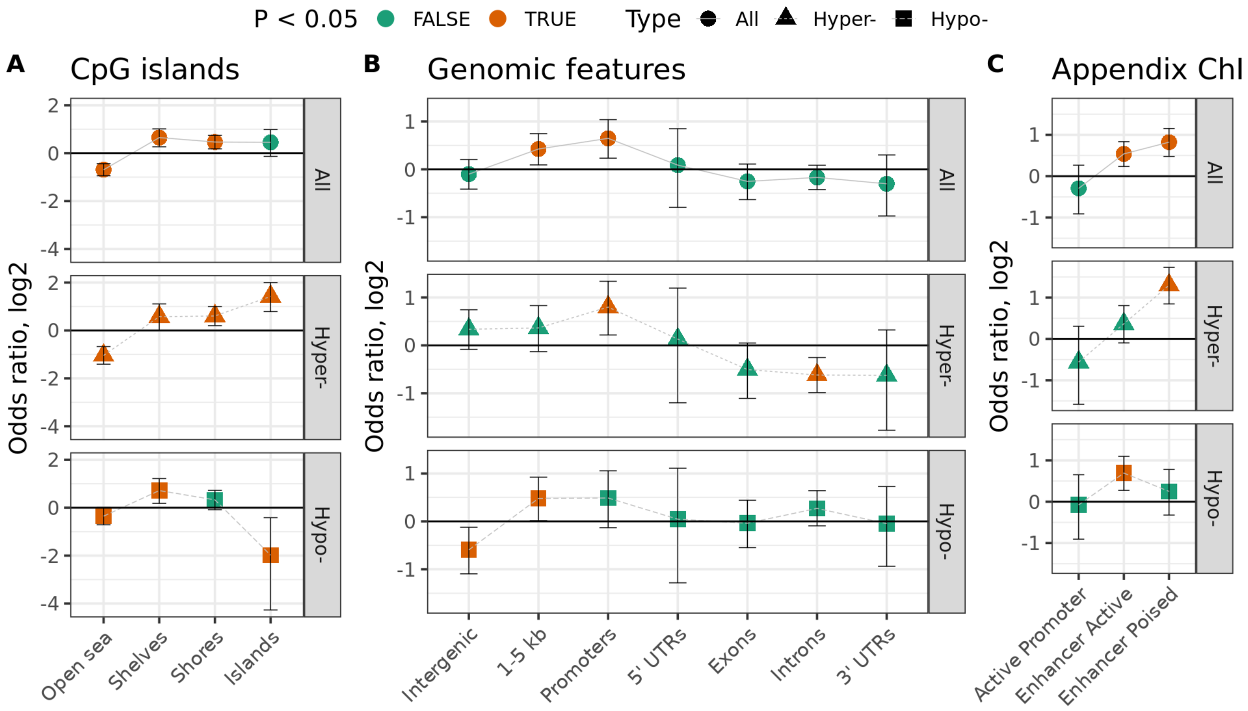

Supplementary Figure 18. Genomic elements enriched by cytosines differentially methylated with age in the appendix of healthy controls restricted to at least 62 years of age. (**A**, **B**, and **C**) Genomic elements enriched with age affected cytosines (All, upper row), hypermethylated cytosines (Hyper-, middle row), and hypomethylated cytosines (Hypo-, lower row) (n = 51 individuals). CpG island and genomic features determined by Bioconductor annotatr package [130] and active/poised enhancers and promoters in the appendix determined by ChIP-seq of H3K4me1 and H3K27ac chromatin marks in appendix. Orange circles represent $p < 0.05$, two-sided Fisher's exact test. Error bars indicate 95% confidence intervals.

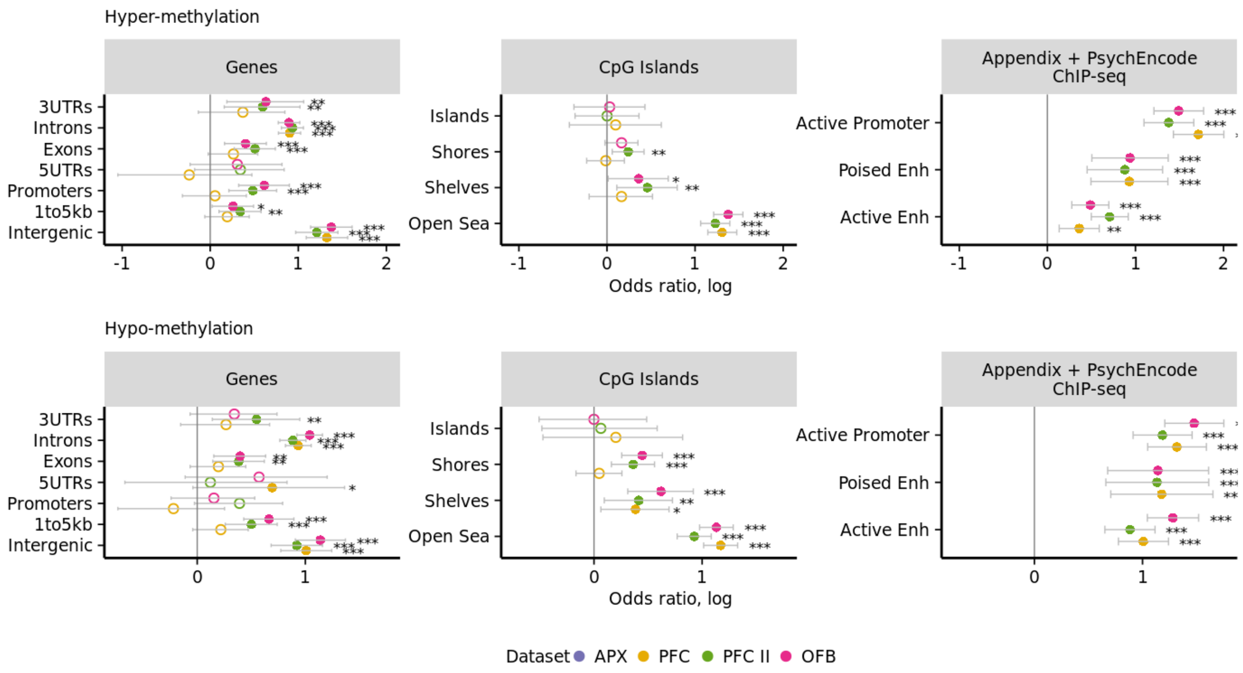

Supplementary Figure 19. Genomic elements exhibiting similar changes in DNA methylation in the PD brain and PD appendix stratified by the direction of methylation change. For each category, the overlap of genomic elements with differentially methylated cytosines between PD appendix and brain datasets was determined. Poised and active enhancers, and active promoters were identified using appendix ChIP-seq (*n* = 3 individuals each for H3K27ac and H3K4me1) and prefrontal cortex (*n* = 9 individuals, PsychENCODE data). Filled circles represent $*p < 0.05$, $**p < 0.01$, and $***p < 0.001$, two-sided Fisher's exact test examining overlap with PD appendix. Error bars indicate 95% confidence intervals. Actual p values are reported in Supplementary Data 21.

Supplementary Table 1. Fold change and percent change of ALP proteins of epigenetically dysregulated genes in the PD appendix. ALP protein changes in the PD appendix and prefrontal cortex shown.

Gene	Accession	Log_2 Fold Change, Appendix	% Change, Appendix	Log_2 Fold Change, Prefrontal cortex	% Change, Prefrontal cortex
ALDOB	P05062	1.30	+ 146 %	0.55	+ 46 %
ANXA11	P50995	– 0.56	– 32 %	n/a	
ASS1	P00966	– 0.33	– 20 %	n/a	
CANX	P27824-2	– 1.81	– 71 %	n/a	
CAPSN1	A0A0C4DGQ5	– 0.69	– 38 %	0.23	+ 17 %
CAT	P04040	0.27	+ 21 %	– 0.25	– 16 %
CLTA	P09496	– 0.49	– 29 %	n/a	
CLTC	A0A087WVQ6	– 0.56	– 32 %	n/a	
COL6A1	P12109	0.39	+ 31 %	n/a	
CTSD	P07339	– 0.50	– 29 %	0.22	+ 16 %
CTSG	P08311	– 0.87	– 45 %	n/a	
CTSZ	Q9UBR2	– 0.35	– 22 %	– 0.24	– 15 %
EEF2	P13639	– 0.77	– 41 %	n/a	
GAA	P10253	– 0.28	– 18 %	n/a	
GABARAPL2	P60520	1.21	+ 131 %	0.44	+ 36 %
GGH	Q92820	– 0.48	– 28 %	0.71	+ 64 %
GNAI2	P04899-4	– 0.60	– 34 %	n/a	
GNS	F6S8M0	– 0.44	– 26 %	0.44	+ 36 %
GPNMB	Q14956	– 0.39	– 24 %	n/a	
HSP90AB1	P08238	– 1.91	– 73 %	– 1.89	– 73 %
HSPA5	P11021	– 0.49	– 29 %	n/a	
HSPA8	P11142	– 0.29	– 18 %	– 0.88	– 46 %

ITGB1	P05556	0.23	+ 17 %	n/a	
MPO	P05164-3	– 0.26	– 16 %	n/a	
NAGA	P17050	– 0.58	– 33 %	0.40	+ 32 %
NAMPT	P43490	– 1.93	– 74 %	– 0.92	– 47 %
PRDX6	P30041	0.35	+ 27 %	n/a	
RAB1A	P62820	– 1.96	– 74 %	0.45	+ 37 %
RNASET2	A0A087WZM2	– 1.27	– 59 %	– 0.26	– 16 %
SERPINA1	P01009	– 0.89	– 46 %	– 0.69	– 38 %
SNCA	P37840	0.87	+ 83 %	0.74	+ 67 %
STX7	O15400	0.39	+ 31 %	0.31	+ 24 %
USP5	P45974	– 0.57	– 33 %	n/a	
VPS35	Q96QK1	– 1.70	– 69 %	– 1.14	– 55 %
