# Supplementary material for: Epigenetic inactivation of the autophagy–lysosomal system in appendix in Parkinson’s disease
Source: Nat Commun. 2021 Aug 26;12:5134. doi: 10.1038/s41467-021-25474-x (PMC8390554; doi:10.1038/s41467-021-25474-x)
Supplement: Supplementary file 3 — Description of Additional Supplementary Files [file 41467_2021_25474_MOESM3_ESM.pdf]

Description of Additional Supplementary Files

Supplementary Data 1: DNA methylation changes in the appendix of PD patients relative to controls.

Supplementary Data 2: Differentially expressed ALP genes in the appendix of PD patients relative to controls.

Supplementary Data 3: DNA methylation changes in the olfactory bulb of PD patients relative to controls.

Supplementary Data 4: DNA methylation changes in the prefrontal cortex neurons of PD patients relative to controls, primary cohort.

Supplementary Data 5: DNA methylation changes in prefrontal cortex neurons with PD Braak stage.

Supplementary Data 6: DNA methylation changes in the prefrontal cortex neurons of PD patients relative to controls, replication cohort.

Supplementary Data 7: ALP genes consistently epigenetically disrupted across the PD appendix and brain.

Supplementary Data 8: Quantitative proteomics analysis of the PD appendix.

Supplementary Data 9: Quantitative proteomic analysis of the PD prefrontal cortex.

Supplementary Data 10:TMT quantitative proteomic analysis of the PD appendix.

Supplementary Data 11: DNA methylation changes with age in appendix of healthy individuals and PD patients.

Supplementary Data 12: DNA methylation changes with age in prefrontal cortex neurons of healthy individuals and PD patients.

Supplementary Data 13: DNA methylation changes with age in olfactory bulb of healthy individuals and PD patients.

Supplementary Data 14: ALP genes consistently epigenetically altered with age in the healthy appendix and healthy prefrontal cortex neurons.

Supplementary Data 15: DNA methylation changes in the cecal patch in response to gut inflammation: wild-type and A30P α-syn mice chronically treated with DSS or water.

Supplementary Data 16: DNA methylation changes in the cecal patch induced by rAAV vector-mediated α-syn aggregation in mice.

Supplementary Data 17: Enrichment of genes with significantly differentially modified cytosines and the dominant direction of modification.

Supplementary Data 18: Demographic and clinical information for human samples.

Supplementary Data 19: Padlock probe libraries and study primers.

Supplementary Data 20: DNA methylation changes with age in appendix of healthy individuals and PD patients restricted to those above 62 years of age.

Supplementary Data 21: Test estimates and p values used to produce figures 1c, 1d, 2c, 4b, 5b, 5c and Supplementary figure 19.
