# Supplementary material for: Epigenetic inactivation of the autophagy–lysosomal system in appendix in Parkinson’s disease
Source: Nat Commun. 2021 Aug 26;12:5134. doi: 10.1038/s41467-021-25474-x (PMC8390554; doi:10.1038/s41467-021-25474-x)
Supplement: Supplementary file 25 — Reporting summary [file 41467_2021_25474_MOESM25_ESM.pdf]

nature research

Corresponding author(s): Juozas Gordevicius

Last updated by author(s): Jun 17, 2021

Reporting Summary

Nature Research wishes to improve the reproducibility of the work that we publish. This form provides structure for consistency and transparency in reporting. For further information on Nature Research policies, see our Editorial Policies and the Editorial Policy Checklist.

Statistics

For all statistical analyses, confirm that the following items are present in the figure legend, table legend, main text, or Methods section.

n/a	Confirmed	
☐	☒	The exact sample size (*n*) for each experimental group/condition, given as a discrete number and unit of measurement
☐	☒	A statement on whether measurements were taken from distinct samples or whether the same sample was measured repeatedly
☐	☒	The statistical test(s) used AND whether they are one- or two-sided<br>*Only common tests should be described solely by name; describe more complex techniques in the Methods section.*
☐	☒	A description of all covariates tested
☐	☒	A description of any assumptions or corrections, such as tests of normality and adjustment for multiple comparisons
☐	☒	A full description of the statistical parameters including central tendency (e.g. means) or other basic estimates (e.g. regression coefficient) AND variation (e.g. standard deviation) or associated estimates of uncertainty (e.g. confidence intervals)
☐	☒	For null hypothesis testing, the test statistic (e.g. *F*, *t*, *r*) with confidence intervals, effect sizes, degrees of freedom and *P* value noted<br>*Give P values as exact values whenever suitable.*
☒	☐	For Bayesian analysis, information on the choice of priors and Markov chain Monte Carlo settings
☐	☒	For hierarchical and complex designs, identification of the appropriate level for tests and full reporting of outcomes
☐	☒	Estimates of effect sizes (e.g. Cohen's *d*, Pearson's *r*), indicating how they were calculated

Our web collection on statistics for biologists contains articles on many of the points above.

Software and code

Policy information about availability of computer code

Data collection	No software was used for data collection
Data analysis	All software used in the analyses is described in the manuscript. All custom data analyses were performed using R programming language (v 3.6.1).<br><br>Custom code needed for reproduction of all reported statistical results and figures pertaining to DNA methylation, RNA-seq and proteomics analysis is available at http://www.vugene.eu/VAI/pdappendixalp and through Zenodo http://doi.org/10.5281/zenodo.5059957. The following software packages available outside of R were used:<br><br>ppDesigner (v1.1): Generate padlock probes<br>Bcl2fastq2 (v2.19): Convertion of reads to FASTQ format<br>Bismark (v0.17.0): Align and call methylation<br>Trim Galore (v0.4.4): Remove adapters and low quality reads<br>Bedtools (v2.25.0): Bed file filtering<br>CIBERSORT (no version): Perform cell-type deconvolution based on methylation<br>STAR (v2.3.5a): RNAseq mapping<br>Picard (v2.18.21): Mark duplicates<br>Samtools (v1.9): Bam filterring<br>Deeptools (v2.3.1): Signal extraction scaling normalization and QC<br>MACS2 (v2.1.2): Peak calling<br>DAVID (v6.8; https://david.ncifcrf.gov/summary.jsp)<br>Cytoscape (v3.7.1) with EnrichmentMap and AutoAnnotate plug-ins

Proteome Discoverer (v. 2.2.0.388, 2017)
STRING-db version 11
BWA v0.7.15
Summit 6.3

For manuscripts utilizing custom algorithms or software that are central to the research but not yet described in published literature, software must be made available to editors and reviewers. We strongly encourage code deposition in a community repository (e.g. GitHub). See the Nature Research guidelines for submitting code & software for further information.

Data

Policy information about availability of data

All manuscripts must include a data availability statement. This statement should provide the following information, where applicable:

- Accession codes, unique identifiers, or web links for publicly available datasets
- A list of figures that have associated raw data
- A description of any restrictions on data availability

Gene lists belonging to ALP Gene Ontology (GO) pathways were obtained from http://geneontology.org (retrieved on 2019-07-01)
Common SNPs were obtained from 1000 Genomes Project (phase 3 v5a 20130502 release for chr1-chr22, v1b 20130502 for chrX; all populations and European populations)

ALP genes were identified using Human Autophagy Database (http://autophagy.lu) and Human Lysosome Gene Database (http://lysosome.unipg.it). We also added genes from the PDGene website (http://www.pdgene.org/)

Data were generated as part of the PsychENCODE Consortium supported by: U01MH103339, U01MH103365, U01MH103392, U01MH103340, U01MH103346, R01MH105472, R01MH094714, R01MH105898, R21MH102791, R21MH105881, R21MH103877, and P50MH106934 awarded to: Schahram Akbarian (Icahn School of Medicine at Mount Sinai), Gregory Crawford (Duke), Stella Dracheva (Icahn School of Medicine at Mount Sinai), Peggy Farnham (USC), Mark Gerstein (Yale), Daniel Geschwind (UCLA), Thomas M. Hyde (LIBD), Andrew Jaffe (LIBD), James A. Knowles (USC), Chunyu Liu (UIC), Dalila Pinto (Icahn School of Medicine at Mount Sinai), Nenad Sestan (Yale), Pamela Sklar (Icahn School of Medicine at Mount Sinai), Matthew State (UCSF), Patrick Sullivan (UNC), Flora Vaccarino (Yale), Sherman Weissman (Yale), Kevin White (UChicago) and Peter Zandi (JHU).

All sequencing data generated in this study are freely available from the NCBI Gene Expression Omnibus (GEO) database under the accession number GSE135751 [https://www.ncbi.nlm.nih.gov/geo/query/acc.cgi?acc=GSE135751]. The mass spectrometry proteomics data have been deposited to the ProteomeXchange Consortium via the PRIDE [113] partner repository with the dataset identifiers PXD015079[http://central.proteomexchange.org/cgi/GetDataset?ID=PXD015079] and PXD021757[http://central.proteomexchange.org/cgi/GetDataset?ID=PXD021757].

Field-specific reporting

Please select the one below that is the best fit for your research. If you are not sure, read the appropriate sections before making your selection.

- [x] Life sciences
- [ ] Behavioural & social sciences
- [ ] Ecological, evolutionary & environmental sciences

For a reference copy of the document with all sections, see nature.com/documents/nr-reporting-summary-flat.pdf

Life sciences study design

All studies must disclose on these points even when the disclosure is negative.

Sample size	No statistical methods were used to predetermine sample size. Balancing age and sex across sample groups were the only sample filtering criteria. Sample sizes were selected based on the available appendix tissue, the prior publications of our lab and that of others using comparable methods in human post-mortem neurons and mouse studies of neurodegenerative disease: Li et al. (DOI: 10.1038/s41467-019-10101-7) used n = 101 human samples in three groups of no/mild, moderate and severe AD. Marshall et al. (DOI: 10.1038/s41593-020-0690-y) used n = 105 individuals, 57 PD and 48 controls. Manfredsson et al. (doi: 10.1016/j.nbd.2018.01.008) used 14/15 rats per group. Subsequent to our DNA methylation analysis we confirmed that our DNA methylation analysis had sufficient statistical power (Figure S17).<br><br>1) Methylation differences in appendix, controls vs PD: 43 individuals, 19 control, 24 PD<br>2) Transcription differences in appendix, controls vs PD: 28 individuals, 16 control, 12 PD<br>3) Label-free proteomics in appendix, controls vs PD: 6 individuals, 3 control, 3 PD<br>4) iTRAQ-labeled proteomics in appendix, controls vs PD: 10 individuals, 5 control, 5 PD<br>5) Methylation differences in prefrontal cortex neurons, primary cohort, controls vs PD: 96 individuals, 42 controls, 52 PD<br>6) Methylation differences in prefrontal cortex neurons, replication cohort, controls vs PD: 28 individuals, 15 controls, 13 PD<br>7) Methylation differences in olfactory bulb, controls vs PD: 23 individuals, 14 controls, 9 PD<br>8) Label-free proteomics in prefrontal cortex neurons, controls vs PD: 6 individuals, 3 control, 3 PD<br>9) Methylation differences in appendix, aging in control and PD: 75 individuals, 52 control, 24 PD<br>10) Transcription differences in appendix, aging in control and PD: 59 individuals, 47 control, 12 PD<br>11) Methylation differences in prefrontal cortex neurons, primary cohort, aging in control and PD: 96 individuals, 42 controls, 52 PD<br>12) Methylation differences in the gut inflammation mouse model: 40 individuals, 11 wild-type + water, 10 wild-type + DSS, 10 A30P + water,

9 A30P + DSS
13) Methylation differences in the rAAV-α-syn overexpression mouse model: 10 individuals, 5 rAAV-GFP, 5 rAAV-α-syn

Data exclusions

Data exclusion criteria was set prior to sample analysis. PCA of all datasets was computed and sample projection onto the first two principal components was plotted, which served to identify outlying samples. Samples deviating from the center of either of the first two principal components by more than 3 standard deviations were deemed outliers and removed from further analysis. Number of samples removed in each analysis:

1) Methylation differences in appendix, controls vs PD: 1 outlying sample was removed
2) Transcription differences in appendix, controls vs PD: 0 outlying samples were removed
3) Label-free proteomics in appendix, controls vs PD: 0 outlying samples were removed
4) iTRAQ-labeled proteomics in appendix, controls vs PD: 0 outlying samples were removed
5) Methylation differences in prefrontal cortex neurons, primary cohort, controls vs PD: 3 outlying samples were removed
6) Methylation differences in prefrontal cortex neurons, replication cohort, controls vs PD: 2 outlying samples were removed
7) Methylation differences in olfactory bulb, controls vs PD: 1 outlying samples were removed
8) Label-free proteomics in prefrontal cortex neurons, controls vs PD: 0 outlying samples were removed
9) Methylation differences in appendix, aging in control and PD: 2 outlying samples were removed
10) Transcription differences in appendix, aging in control and PD: 0 outlying samples were removed
11) Methylation differences in prefrontal cortex neurons, primary cohort, aging in control and PD: 3 outlying samples were removed
12) Methylation differences in the gut inflammation mouse model: 0 outlying samples were removed
13) Methylation differences in the rAAV-α-syn overexpression mouse model: 0 outlying samples were removed

Replication

Technical replicates were included in DNA methylation (bisulfite padlock probe) sequencing experiments. The human appendix dataset had 16 technical replicates, mouse datasets included 7 technical replicates. Prefrontal cortex neuron primary cohort dataset had 4 technical replicates. Olfactory bulb dataset included 3 technical replicates. We confirmed that technical reproducibility exceeded biological variability in the human and mouse datasets by comparing mean pairwise correlation between biological and technical replicates.

Randomization

All samples randomized during isolation and library preparation. Samples were only identified during the statistical analysis to prevent any bias.

Blinding

All samples de-identified during isolation and library preparation. Samples were only identified during the statistical analysis to prevent any bias. Immunohistochemistry and behavioral studies in mice were conducted by an experimenter blind to genotype and treatment.

Reporting for specific materials, systems and methods

We require information from authors about some types of materials, experimental systems and methods used in many studies. Here, indicate whether each material, system or method listed is relevant to your study. If you are not sure if a list item applies to your research, read the appropriate section before selecting a response.

Materials & experimental systems

n/a	Involved in the study	
☐	☒	Antibodies
☒	☐	Eukaryotic cell lines
☒	☐	Palaeontology and archaeology
☐	☒	Animals and other organisms
☐	☒	Human research participants
☒	☐	Clinical data
☒	☐	Dual use research of concern

Methods

n/a	Involved in the study	
☐	☒	ChIP-seq
☐	☒	Flow cytometry
☒	☐	MRI-based neuroimaging

Antibodies

Antibodies used

NeuN Alexa Fluor 488, Abcam, product number: ab190195, concentration used: 1:500, host species: Rabbit
HuC/HuD Monoclonal Antibody (16A11), Thermo Fisher Scientific, catalog number: A-21271, concentration used: 1:2000, host species: mouse
Purified Mouse Anti-α-Synuclein, BD Biosciences, catalog number: 610786, concentration used: 1:1000, host species: mouse
Anti-Alpha-synuclein (phospho S129) antibody [P-syn/81A], Abcam, product number: ab184674, concentration used: 1:10,000, host species: mouse
goat anti-mouse IgG2a Alexa Fluor 488, Thermo Fisher Scientific, catalog number: A-21131 , concentration used: 1:500
goat anti-mouse IgG1 Alexa Fluor 488, Thermo Fisher Scientific, catalog number: A-21121, concentration used: 1:500
goat anti-mouse IgG2b Alexa Fluor 594, Thermo Fisher Scientific, catalog number: A-21145, concentration used: 1:500
anti-histone H3 acetyl K27 antibody, Abcam, product number: ab4729), concentration used: 1:40
anti-histone H3 mono methyl K4 antibody, Abcam, product number: ab8895, concentration used: 1:40
Normal Rabbit IgG, Cell Signaling, product number: 2729 , concentration used: 1:40

Validation	NeuN Alexa Fluor 488 has been validated for flow cytometry 1:500 concentration (http://www.abcam.com/neun-antibody-epr12763-neuronal-marker-alexa-fluor-488-ab190195.html)<br><br>HuC/HuD antibody has been shown to specifically label neuronal cells in zebrafish, chick, canaries, and humans, and is likely to label neuronal cells in most vertebrate species, and has been used for Immunofluorescence (IF) in mice in 8 publications according to the manufacturer's website (https://www.thermofisher.com/antibody/product/HuC-HuD-Antibody-Monoclonal/A-21271)<br><br>Purified Mouse Anti-α-Synuclein antibody was tested for immunofluorescence and reactivity in mouse during development according to the manufacturer's website (https://www.bdbiosciences.com/eu/applications/research/stem-cell-research/ectoderm-markers/human/purified-mouse-anti--synuclein-42-synuclein/p/610787)<br><br>Anti-Alpha-synuclein (phospho S129) antibody [P-syn/81A] has been used for IHC in mice in the following publications:<br><br>Du T et al. Injection of a-syn-98 Aggregates Into the Brain Triggers a-Synuclein Pathology and an Inflammatory Response. Front Mol Neurosci 12:189 (2019).<br><br>Uemura N et al. Inoculation of a-synuclein preformed fibrils into the mouse gastrointestinal tract induces Lewy body-like aggregates in the brainstem via the vagus nerve. Mol Neurodegener 13:21 (2018)<br><br>According to the manufacturer, all Anti-Mouse secondary antibodies are affinity-purified antibodies with well-characterized specificity for mouse immunoglobulins and are useful in the detection, sorting or purification of its specified target, and are cited in multiple publications

Animals and other organisms

Policy information about studies involving animals; ARRIVE guidelines recommended for reporting animal research

Laboratory animals	Gut inflammation studies were conducted in Wild type mice C57BL/6 mice and hemizygous Tg(Thy1-SNCA*A30P)18Pjk mice (A30P α-syn). A30P α-syn mice express human α-syn with the A30P mutation under the neuron selective Thy1 promoter (Kahle, P.J., et al., Subcellular localization of wild-type and Parkinson's disease-associated mutant alpha -synuclein in human and transgenic mouse brain. J Neurosci, 2000. 20(17): p. 6365-73.). A30P α-syn mice have been maintained on a C57BL/6 background for more than 10 generations. Wild-type and A30P α-syn mice were exposed to a chronic DSS protocol at 3 months of age. Mice were then given a 4-week long recovery period during which they received normal drinking water, followed by tissue harvest.<br><br>Approximately equal numbers of male and female mice were used. To the extent possible, littermates were used in the experiments. The mice were generated and kept in filter cover cages under normal housing conditions with a 12h dark/light cycle (light from 6am to 6pm), constant ambient temperature at 22°C (+/- 2°C) and air humidity ranging from 40%-60%.<br><br>rAAV a-syn overexpression experiments were conducted with Male C57BL/6J mice obtained from Jackson Labs at 8 weeks of age. Animals were housed in a 12 hour light (on 6am to 6pm). Ambient temperature is 69.9 to 73F with a humidity ranging from 0% to 52%.
Wild animals	No wild animals were involved in this study
Field-collected samples	No field-collected samples were involved in this study
Ethics oversight	The gut inflammation animal experiments were endorsed by a Roche internal review board and approved by the local animal welfare authorities in Canton Basel-Stadt, Basel, Switzerland.<br><br>The experiment using rAAV vectors in mice were performed in accordance and with ethical approval of the Michigan State University Institutional Animal Care & Use Committee guidelines (AUF 08-16-148-00).

Note that full information on the approval of the study protocol must also be provided in the manuscript.

Human research participants

Policy information about studies involving human research participants

Population characteristics	Postmortem appendix tissue from PD patients and controls was obtained from the Oregon Brain Bank. In our aging analysis, we included surgically-isolated, histologically normal appendix tissue from control (non-PD) individuals obtained from the Spectrum Health Universal Biorepository and Cooperative Human Tissue Network (CHTN). Appendix surgical samples were from individuals undergoing a right hemicolectomy for intestinal cancer not involving the appendix (appendix incidentally removed and histologically confirmed to be normal). Prefrontal cortex tissue was obtained from the NIH NeuroBioBank, Parkinson's UK Brain Bank, Michigan Brain Bank (primary cohort), or the Oregon Brain Bank (replication cohort). Olfactory bulb tissue was obtained from the Oregon Brain Bank. For the study samples, we had information on demographics (age, sex), tissue quality (postmortem/surgical interval), and pathological staging (Supplementary Data 15). Appendix, prefrontal cortex, and olfactory bulb postmortem tissue from PD patients have evident brain Lewy pathology (PD Braak stages III-VI), whereas control individuals have no Lewy pathology in the brain. Sample information is detailed in Supplementary Data 18.

	The following covariates were used in our statistical analysis:<br><br>1) Methylation differences in appendix, controls vs PD: Diagnosis, age, gender, PMI, Cohort, SmartSVA vectors<br>2) Transcription differences in appendix, controls vs PD: Diagnosis, age, gender, PMI, RIN, SmartSVA vectors<br>3) Label-free proteomics in appendix, controls vs PD: Diagnosis<br>4) iTRAQ-labeled proteomics in appendix, controls vs PD: Diagnosis, age, gender, PMI<br>5) Methylation differences in prefrontal cortex neurons, primary cohort, controls vs PD: Diagnosis, Age, Gender, PMI, Neuronal proportion<br>6) Methylation differences in prefrontal cortex neurons, replication cohort, controls vs PD: Diagnosis, Age, Gender, PMI, SmartSVA vectors<br>7) Methylation differences in olfactory bulb, controls vs PD: Diagnosis, Age, Gender, PMI<br>8) Label-free proteomics in prefrontal cortex neurons, controls vs PD: Diagnosis<br>9) Methylation differences in appendix, aging in control and PD: Diagnosis, Age, Gender, PMI, Cohort<br>10) Transcription differences in appendix, aging in control and PD: Diagnosis, Age, Gender, Cohort, PMI, RIN, SmartSVA vectors<br>11) Methylation differences in prefrontal cortex neurons, primary cohort, aging in control and PD: Diagnosis, Age, Gender, PMI, Neuronal proportion<br>12) Methylation differences in the gut inflammation mouse model: Genotype, Treatment, Sex<br>13) Methylation differences in the rAAV-α-syn overexpression mouse model: Treatment
Recruitment	At the time of tissue request from the brain banks we requested that samples be over the age of 50 with a postmortem interval of less than 48 h, and that they were age and sex balanced between PD patients and controls. No other prior selection criteria was used for our cohort. No other population data, such as race, was known to us, however, we used computational tools such as RUV to account for any unwanted variability in the data.
Ethics oversight	For the human tissue studies, the study protocol was ethically approved by the institutional review board at the Van Andel Research Institute (IRB #15025).

Note that full information on the approval of the study protocol must also be provided in the manuscript.

ChIP-seq

Data deposition

☒ Confirm that both raw and final processed data have been deposited in a public database such as GEO.

☒ Confirm that you have deposited or provided access to graph files (e.g. BED files) for the called peaks.

Data access links<br>*May remain private before publication.*	The following secure token has been created to allow review of record GSE135751 while it remains in private status: obgfeyaydrgxlkv. https://www.ncbi.nlm.nih.gov/geo/query/acc.cgi?acc=GSE135751
Files in database submission	GSE135750_ACvsinput_peaks.narrowPeak_IDR0.05_conservative_final.bed<br>GSE135750_ACvsinput_peaks.txt<br>GSE135750_MEvsinput_peaks.narrowPeak_IDR0.05_conservative_final.bed<br>GSE135750_MEvsinput_peaks.txt<br>GSE135750_RAW.tar
Genome browser session<br>(e.g. UCSC)	No longer aplicable

Methodology

Replicates	Chromatin immunoprecipitation (ChIP) was used to identify active enhancers and promoters in the healthy human appendix (n = 3 individuals).
Sequencing depth	Libraries for appendix ChIP (H3K27ac, H3K4me1) and input samples were prepared by the Van Andel Genomics Core from 10 ng of input material and all available immunoprecipitated material using the KAPA Hyper Prep Kit (v5.16) (Kapa Biosystems). Prior to PCR amplification, end-repaired and A-tailed DNA fragments were ligated to IDT for Illumina TruSeq UD Indexed Adapters (Illumina Inc.). The quality and quantity of the finished libraries were assessed using a combination of Agilent DNA High Sensitivity chip (Agilent Technologies, Inc.), and QuantiFluor dsDNA System (Promega Corp.). Sequencing (single-end 100 bp) was performed on an Illumina NovaSeq6000 sequencer producing ~99.6 million reads per sample. Base calling was done by Illumina RTA3, and output was demultiplexed and converted to FASTQ format with Illumina Bcl2fastq2 v2.19.
Antibodies	To perform the ChIP, 200 µl of sheared DNA was exposed to 5 µg of anti-histone H3 acetyl K27 antibody (Abcam #ab4729), anti-histone H3 mono methyl K4 antibody (Abcam #ab8895), or control IgG (Cell Signaling).
Peak calling parameters	Adapter sequence from raw sequencing reads were removed using Trim Galore (v0.4.4). Sequenced reads from H3K27ac and H3K4me1 immunoprecipitated samples and matched input controls from human appendix were mapped to the human reference genome (GRCh37/hg19) with BWA (v0.7.15) [129]. A combination of Picard and Samtools (v1.9) was used to mark and remove PCR duplicates, respectively. Deeptools (v2.3.1) [130] was used for quality controls and narrow peaks were called using MACS2 [131] for each sample.

Data quality	Consensus peaks were called using MACS2 [131] combined peak calls (removing blacklist regions) and IDR, following ENCODE ChIP-seq guidelines [132], which yielded 26,381 H3K27ac peaks and 37,727 H3K4me1 peaks.
Software	No additional software or custom code were used.

Flow Cytometry

Plots

Confirm that:

- [x] The axis labels state the marker and fluorochrome used (e.g. CD4-FITC).
- [x] The axis scales are clearly visible. Include numbers along axes only for bottom left plot of group (a 'group' is an analysis of identical markers).
- [x] All plots are contour plots with outliers or pseudocolor plots.
- [ ] A numerical value for number of cells or percentage (with statistics) is provided.

Methodology

Sample preparation	Isolation of neuronal nuclei from prefrontal cortex was performed using a flow cytometry-based approach, similarly to previously described. Briefly, human brain tissue (~250mg) for each sample was minced in 2mL PBSTA (0.3M sucrose, 1X phosphate buffered saline (PBS), 0.1% Triton X-100) and homogenized in PreCellys CKMix tubes with a Minilys (3,000rpm for 5 s, 5min on ice, 3 times) (Bertin Instruments). Samples homogenates were filtered through Miracloth (EMD Millipore), rinsed with 2mL of PBSTA and placed on a sucrose cushion (1.4M sucrose). Nuclei were pelleted by centrifugation at 4000×g for 30min 4°C using a swinging bucket rotor and the pellet was incubated in 700µl of 1X PBS on ice for 20min. The nuclei were then gently resuspended and blocking mix (100µl of 1X PBS with 0.5% BSA (Thermo Fisher Scientific), and 10% normal goat serum (Gibco)) was added to each sample. Anti-NeuN antibody with Alex Fluor 488 (1:500; Abcam; ab190195) was added and samples were incubated 45min at 4°C with gentle mixing. Immediately prior to flow cytometry sorting, nuclei were stained with 7-AAD (Thermo Fisher Scientific) and passed through a 30 µM filter (SystemX).
Instrument	MoFlo Astrios (Beckman Coulter)
Software	Summit 6.3
Cell population abundance	Approximately 1 million NeuN+ nuclei were sorted for each sample with an average purity of 97.38% ± 0.33%.
Gating strategy	Starting population was defined using FSC and SSC. Singlets were then gated followed by 7AAD isolation of positive nuclei. NeuN positive nuclei (neuronal) were then gated.

- [x] Tick this box to confirm that a figure exemplifying the gating strategy is provided in the Supplementary Information.
